# MRSA Clonal Complex 22 Strains Harboring Toxic Shock Syndrome Toxin (TSST-1) Are Endemic in the Primary Hospital in Gaza, Palestine

**DOI:** 10.1371/journal.pone.0120008

**Published:** 2015-03-17

**Authors:** Nahed Al Laham, José R. Mediavilla, Liang Chen, Nahed Abdelateef, Farid Abu Elamreen, Christine C. Ginocchio, Denis Pierard, Karsten Becker, Barry N. Kreiswirth

**Affiliations:** 1 Department of Laboratory Medicine, Al Azhar University-Gaza, Gaza Strip, Palestine; 2 Public Health Research Institute, Rutgers University, Newark, New Jersey, United States of America; 3 The Central Laboratories, Ministry of Health, Gaza Strip, Palestine; 4 Department of Pathology and Laboratory Medicine, North Shore-LIJ Health System, Lake Success, New York, United States of America; 5 Hofstra North Shore-LIJ School of Medicine, Hempstead, New York, United States of America; 6 Institute of Medical Microbiology, University Hospital of Brussels, Brussels, Belgium; 7 Institute of Medical Microbiology, University Hospital of Muenster, Muenster, Germany; Ross University School of Veterinary Medicine, SAINT KITTS AND NEVIS

## Abstract

**Background:**

Methicillin-resistant *Staphylococcus aureus* (MRSA) is an important pathogen in both community and healthcare-related settings worldwide. Current knowledge regarding the epidemiology of *S*. *aureus* and MRSA in Gaza is based on a single community-based carriage study. Here we describe a cross-sectional analysis of 215 clinical isolates collected from Al-Shifa Hospital in Gaza during 2008 and 2012.

**Methods:**

All isolates were characterized by *spa* typing, SCC*mec* typing, and detection of genes encoding Panton-Valentine leukocidin (PVL) and toxic shock syndrome toxin (TSST-1). Representative genotypes were also subjected to multilocus sequence typing (MLST). Antibiotic susceptibility testing was performed using VITEK2 and MicroScan.

**Results:**

MRSA represented 56.3% of all *S*. *aureus* strains, and increased in frequency from 2008 (54.8%) to 2012 (58.4%). Aside from beta-lactams, resistance was observed to tetracycline, erythromycin, clindamycin, gentamicin, and fluoroquinolones. Molecular typing identified 35 *spa* types representing 17 MLST clonal complexes (CC), with *spa* 998 (Ridom t223, CC22) and *spa* 70 (Ridom t044, CC80) being the most prevalent. SCC*mec* types I, III, IV, V and VI were identified among MRSA isolates, while type II was not detected. PVL genes (*lukF/S-PV*) were detected in 40.0% of all isolates, while the TSST-1 gene (*tst*) was detected in 27.4% of all isolates, with surprisingly high frequency within CC22 (70.4%). Both PVL and TSST-1 genes were found in several isolates from 2012.

**Conclusions:**

Molecular typing of clinical isolates from Gaza hospitals revealed unusually high prevalence of TSST-1 genes among CC22 MRSA, which is noteworthy given a recent community study describing widespread carriage of a CC22 MRSA clone known as the ‘Gaza strain’. While the latter did not address TSST-1, *tst*-positive *spa* 998 (Ridom t223) has been detected in several neighboring countries, and described as endemic in an Italian NICU, suggesting international spread of a ‘Middle Eastern variant’ of pandemic CC22 strain EMRSA-15.

## Introduction

*Staphylococcus aureus* is one of the most prevalent human pathogens isolated from hospitalized patients worldwide, and its importance in community settings continues to increase. *S*. *aureus* causes a broad variety of diseases ranging from skin and soft-tissue infections to bacteremia, osteomyelitis, infective endocarditis, and necrotizing pneumonia [1–3]. In recent decades, MRSA has emerged as the most frequently identified antibiotic-resistant pathogen in many parts of the world, including North Africa and the Middle East [4]. While hospital-acquired MRSA (HA-MRSA) strains remain endemic in most of these regions, in recent years community-acquired (CA-MRSA) strains have emerged as a cause of invasive and life-threatening infections in young, healthy patients with no significant healthcare exposure [2,5–8]. Within the past few years, livestock-associated MRSA (LA-MRSA) have posed an additional threat [9–11]. Fortunately, whereas HA-MRSA isolates are generally multi-drug resistant, CA-MRSA and LA-MRSA tend to be resistant primarily to beta-lactam antibiotics and, in the case of LA-MRSA CC398, to tetracycline as well.

Molecular typing techniques are indispensable for understanding the evolution and epidemiology of *S*. *aureus* and MRSA. The most widely used techniques include staphylococcal protein A (*spa*) typing [12,13], staphylococcal cassette chromosome (SCC) *mec* typing [14], multilocus sequence typing (MLST) [15], and pulsed-field gel electrophoresis (PFGE) [16]. Other markers of interest include virulence factors such as Panton-Valentine leukocidin (PVL) and TSST-1, the principal cause of staphylococcal toxic shock syndrome [17]. Approximately 20% of *S*. *aureus* isolates possess the gene encoding TSST-1 (*tst*) [18], which is harbored by a family of mobile staphylococcal pathogenicity islands (SaPI) [19–21]. The distribution of TSST-1 appears limited to a handful of clonal lineages, and is most frequently associated with methicillin-susceptible *S*. *aureus* (MSSA) strains belonging to MLST clonal complex (CC) 30. More recently, a *tst*-positive strain of CC5 MRSA has been documented in France [22], while several reports have described *tst*-positive CC22 MRSA strains in Abu Dhabi [23], Jordan [24,25], Kuwait [26], Saudi Arabia [27], India [3], Italy [28,29], the United Kingdom [30], and the United States [31]. Several recent studies have also described *tst*-positive CC30 [25] and CC80 [24,32] MRSA in Jordan.

Molecular epidemiological data about *S*. *aureus* and MRSA in the Middle East, including the Palestinian Territories, are generally scarce and insufficient [4,33–35], with current knowledge regarding the epidemiology of *S*. *aureus* in Gaza based on a single recent community-based carriage study [36]. The Gaza Strip (geographic coordinates 31°25′ N, 34° 20′ E) is a narrow territory (41 km long and 6–12 km wide) along the eastern Mediterranean coast, with tightly-controlled borders abutting Israel and the Sinai Peninsula of Egypt. It is considered one of the most densely populated areas in the world, with a population of about 1.7 million inhabitants (37.5% of the total estimated Palestinian population), and a population density (4,073/km^2^) nearly ten-fold greater than that of the West Bank (433/km^2^) [37]. Over the last decade, the socioeconomic situation in Gaza has declined steadily, leaving nearly 80% of the population dependent on international assistance. The healthcare infrastructure within Gaza suffers accordingly from the long-term effects of war, economic isolation, and border closures, with marked scarcity of medical equipment and instrumentation. Recent events such as war, border fighting and regional political unrest have only served to exacerbate the situation [38].

Here we describe the molecular characteristics and antibiotic susceptibilities of clinical MRSA and MSSA isolates collected from the largest medical complex and primary hospital in Gaza (Al-Shifa Hospital) in 2008 and 2012. To the best of our knowledge, this is the first report describing clinical *S*. *aureus* infection within the Gaza Strip. We note the high prevalence of a *tst*-positive CC22 MRSA clone, which is likely related to the ‘Gaza strain’ described in the aforementioned community-based carriage study [36], as well as the ‘Middle Eastern variant’ of EMRSA-15 from a recent NICU outbreak in Sicily, Italy [28].

## Methods

### Bacterial isolates

For the present study, 215 unique *S*. *aureus* isolates associated with diverse clinical infections were collected from the largest public tertiary referral hospital (Al-Shifa Hospital) in Gaza City. Of the 215 unique *S*. *aureus* isolates, 126 were collected in 2008, out of a total unique 1091 bacterial isolates (March 1–July 31); while 89 were collected in 2012, out of a total unique 1121 bacterial isolates (March 1–August 11). Isolates were obtained directly from the clinical laboratory of Al-Shifa Hospital, and represent complete capture of all *S*. *aureus* isolates during the stated collection periods. Although a defined sampling strategy was not employed, the collected strains likely reflect the clinical epidemiology of *S*. *aureus* in Gaza, since Al-Shifa Hospital is the primary referral hospital for patients from all areas of the Gaza Strip.

### Identification of *S*. *aureus* and MRSA

*S*. *aureus* isolates were identified phenotypically using one or more of the following methods: catalase test, tube coagulase test, Pastorex Staph Plus latex agglutination (Bio-Rad, Hercules, California), and the Staph ID 32 API system and VITEK 2 Compact Gram Positive Card (bioMérieux, Paris, France). Multiplex PCR was also used for detection of the *nuc* (*S*. *aureus* species-specific) and *mecA* (methicillin-resistance) genes [39,40]. DNA for molecular typing was isolated as described previously, with different methods used for the 2008 [18] and 2012 [41] isolates.

### Antimicrobial susceptibility testing

Antibiotic susceptibility testing for the 2008 isolates was performed with the automated VITEK 2 Compact automated system, using the VITEK 2 Compact Gram Positive Card (bioMérieux, Paris, France); antimicrobials tested included amoxicillin/clavulanic acid, ampicillin, ampicillin/sulbactam, azithromycin, cefaclor, cefotaxime, ceftriaxone, cefuroxime, clarithromycin, clindamycin, erythromycin, fosfomycin, fusidic acid, gentamicin, imipenem, levofloxacin, linezolid, moxifloxacin, mupirocin, nitrofurantoin, oxacillin, penicillin, rifampin, teicoplanin, tetracycline, tigecycline, tobramycin, trimethoprim/sulfamethoxazole, and vancomycin. Susceptibility testing for the 2012 isolates was performed using the MicroScan WalkAway Plus System (Siemens Healthcare, Erlangen, Germany); antimicrobials tested included amoxicillin/clavulanic acid, ampicillin, ampicillin/sulbactam, cefazolin, cefoxitin, ciprofloxacin, clindamycin, daptomycin, erythromycin, gentamicin, levofloxacin, linezolid, moxifloxacin, oxacillin, penicillin, rifampin, synercid, tetracycline, trimethoprim/sulfamethoxazole, and vancomycin. Due to the different selection of antimicrobials in each panel, only antibiotics common to each system were compared in this report.

### Molecular typing

All 215 *S*. *aureus* isolates were characterized by staphylococcal protein A (*spa*) typing [12]; 2008 isolates were *spa* typed using the Ridom StaphType software [13,42], while 2012 isolates were *spa* typed using eGenomics software [12,41], with Ridom assignments made using the Spa Server website (http://spa.ridom.de/). In order to avoid confusion, both designations are used throughout this manuscript, with eGenomics *spa* types prefaced by the term “*spa*”, and Ridom *spa* types shown in parentheses. All MRSA isolates were subjected to staphylococcal cassette chromosome (SCC)*mec* typing; 2012 isolates were typed using multiplex real-time PCR [43], while 2008 isolates were typed using both multiplex conventional [44] and real-time [43] PCR. Multilocus sequence typing (MLST) was performed as described previously [15] on a representative subset of 42 isolates, with clonal complexes inferred via eBURST analysis [45]; all other clonal complexes were inferred from *spa* typing data as described previously [41], using both the Ridom Spa Server website and our own internal eGenomics database. Clonal complex sub-groups with distinct genotypic signatures were classified as individual CCs [8], *e*.*g*. ST239 strains were classified as CC239 rather than CC8; ST34 strains were classified as CC34 rather than CC30; and ST291 strains were classified as CC291 rather than CC398.

### Detection of PVL and TSST-1 genes

All isolates were tested for the genes encoding Panton-Valentine leukocidin (PVL) and toxic shock syndrome toxin (TSST-1). The 2008 isolates were initially tested for PVL (*lukS-PV* and *lukF-PV*) and TSST-1 (*tst*) using previously described methods [39,46], then re-tested along with the 2012 isolates as follows. The 2012 isolates were tested for PVL (*lukF-PV*) using a previously described real-time PCR assay [47], and tested for TSST-1 (*tst*) using a novel real-time PCR assay (this study). The novel assay uses the following oligonucleotide sequences: a) forward primer tst.sy-F1, CCCTTTGTTGCTTGCG; b) reverse primer tst.sy-R1 CGTTTGTAGATGCTTTTGC; and c) molecular beacon probe tst-MB, 5′-6-HEX d(cgcgtCCCCTGTTCCCTTATCATCTacgcg)-DABCYL-3′, where stem sequences are underlined, HEX is 6-hexachlorofluorescein, and DABCYL is 4-[4-(dimethylamino)phenylazo]benzoic acid. PCR cycling conditions for the novel assay were as described previously [43].

Production of TSST-1 toxin by 17 representative *tst*-positive strains was assayed via reversed passive latex agglutination (RPLA), using the Oxoid TST-RPLA Toxin Detection Kit from Thermo Fisher Scientific (Oxoid Ltd., United Kingdom), according to the manufacturer’s instructions.

### Sequencing of TSST-1 and SaPI element

Long-range PCR assays were employed to partially amplify the SaPI region of three *tst*-positive isolates. Known SaPI sequences were downloaded from GenBank and used as templates for primer design. Forward primers were designed to target the SaPI integrase gene, while the reverse primer is located within *tst* and is universally conserved among all known SaPIs. Primer oligonucleotide sequences were as follows: a) Int-F1, GCGTGATGTTTAGCGTCTGA (targets the SaPI integrase in strains N315, Mu50, and RN3984); b) Int-F2, TTTGAAGTTTTGCCTCAGCA (targets the SaPI integrase in strains RF122 and ED133); c) Int-F3, TTCCCTACAAGAGATGCAACA (targets the integrases in SaPI68111 and SaPIj50); d) Int-F4, CCACTCATCACTTGCAGCAT) (targets the integrase sequence in *Tn*557); and e) reverse primer Tst-Rv, GTAAGCCCTTTGTTGCTTGC (conserved in all *tst*-harboring SaPI elements). Genomic bacterial DNA was isolated using a previously-described method [48], and long-range PCR was performed using the Advantage GC Genomic LA PCR Polymerase Kit (Clontech Laboratories, Inc., Mountain View, CA), according to the manufacturer’s protocol. The resulting amplicons were then sequenced by primer walking as described previously [48]. One of the three identical SaPI sequences was submitted to GenBank (NCBI) as accession number KP334121.

### Statistical analysis

Comparisons of selected data from 2008 and 2012 were performed by Chi-square analysis using GraphPad Software (San Diego, California). Chi-square tests (two-tailed) with *p*-values ≤ 0.05 were considered statistically significant.

### Ethics Statement

The study protocol and data handling were approved by the ethical committee at the laboratory medicine department of Al Azhar University-Gaza, which does not have an Institutional Review Board (IRB) at this time. The study was performed in accordance with the ethical standards established in the 1964 and 1975 Declaration of Helsinki, and the modifications thereafter.

## Results

### *S*. *aureus* and MRSA prevalence

A total of 215 unique *S*. *aureus* clinical isolates were collected from individual patients. Of these, 126 were collected in 2008 (out of a total 1091 bacterial isolates), while 89 were collected in 2012 (out of a total 1121 bacterial isolates). The majority of the isolates were from skin (44.2%), surgical site (30.7%), urogenital (14.9%), and bloodstream (8.4%) infections. Unfortunately, patient demographic data were very limited, and could not be addressed in this report. *S*. *aureus* represented 9.7% of all bacterial isolates (215/2212), decreasing significantly (*p* = 0.005) in prevalence from 11.5% in 2008 (126/1091) to 7.9% in 2012 (89/1121). MRSA (defined both phenotypically and by the presence of *mecA*) represented 5.5% of all bacterial isolates (121/2212) and 56.3% (121/215) of all *S*. *aureus* strains, with MRSA prevalence increasing from 54.8% in 2008 (69/126) to 58.4% in 2012 (52/89).

### Antimicrobial resistance

**Fig 1:**
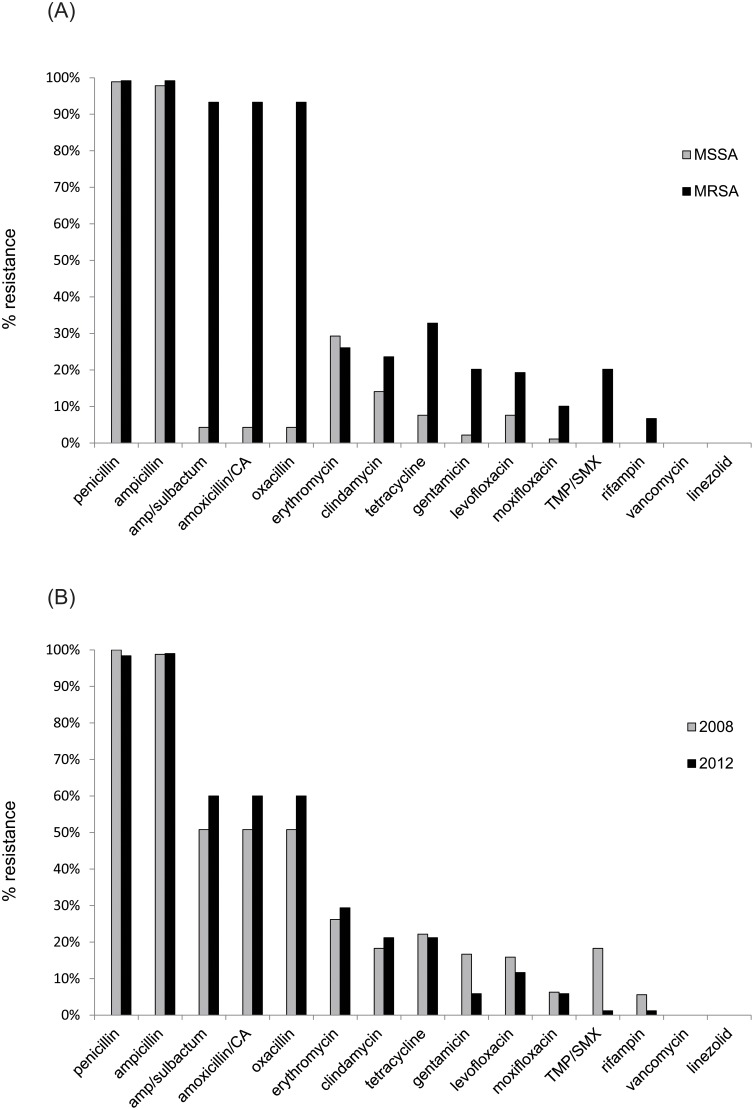
Antimicrobial resistance frequencies among *S*. *aureus* isolates in this study. Vertical bars denote relative resistance to selected antimicrobials described in the text. a. Black bars denote percent resistance among MRSA isolates; gray bars denote resistance among MSSA isolates. b. Gray bars denote percent resistance among *S*. *aureus* isolates from 2008; black bars denote resistance among *S*. *aureus* isolates from 2012. Only antimicrobials tested in both 2008 (VITEK 2) and 2012 (MicroScan) are depicted. Amp, ampicillin; CA, clavulanic acid; MRSA, methicillin-resistant *Staphylococcus aureus*; MSSA, methicillin-susceptible *Staphylococcus aureus*; TMP-SMX, trimethoprim-sulfamethoxazole.

Resistance to multiple classes of antimicrobials was observed among both MRSA and MSSA, including aminoglycosides, beta-lactams, cephalosporins, fluoroquinolones, fusidic acid, macrolides, and tetracycline. Since different susceptibility testing methods were used in 2008 (VITEK 2) and 2012 (MicroScan), direct comparisons were only possible for 15 of the total 35 antimicrobials tested. Fig. 1 shows the prevalence of resistance to these 15 antimicrobials for: a) MRSA *vs*. MSSA, and b) 2008 *vs*. 2012, respectively. Whereas resistance to beta-lactams increased from 2008 to 2012, resistance to various other classes of antimicrobials decreased, associated primarily with the apparent replacement of several multi-resistant clones by less resistant ones. MSSA strains exhibited nearly complete resistance to penicillin (98.9%) and ampicillin (97.8%), but were mostly susceptible to the remaining beta-lactams. In general, susceptibility of *S*. *aureus* isolates from both 2008 and 2012 was greater than 75% to common anti-staphylococcal agents such as erythromycin, fluoroquinolones, gentamicin, and tetracycline. No resistance was observed to any of the following antibiotics: linezolid and vancomycin (both 2008 and 2012); fosfomycin, mupirocin, nitrofurantoin, teicoplanin, and tigecycline (2008 only); and daptomycin and synercid (2012 only).

### SCC*mec* typing

Among the MRSA isolates, SCC*mec* types I (2.5%), III (7.4%), IV (79.3%), V (7.4%) and VI (3.3%) were identified; SCC*mec* type II was not detected in this collection (Table 1). SCC*mec* type IV increased in prevalence from 75.4% (52/69) in 2008 to 84.6% (44/52) in 2012, with subtypes IVa and IVc accounting for 61.5% (59/96) and 36.5% (35/96) of all SCC*mec* IV isolates, respectively; two SCC*mec* IV isolates could not be sub-typed. SCC*mec* type V was only detected in 2008, while SCC*mec* types I and VI were only detected in 2012. Notably, only the SCC*mec* type III isolate from 2012 appeared to harbor SCC*mercury* (III-Hg), as indicated by the presence of *ccrC* [14,43].

**Table 1:** Molecular characteristics of *S*. *aureus* isolates described in this study.

Clonal complex	Sequence type ^*a*^	*spa* type (Ridom)	*spa* repeats (eGenomics)	*spa* type (eGenomics)	SCC*mec* type ^*b*^	PVL (*lukF*)	TSST-1 (*tst*)	Year of isolation	Clinical source ^*d*^	No. of isolates
CC 1	ST 1278	t3643	ZMBME2JQQQ	1598	-			2008	P, SA, SS, SW	5
	^-^	t3643	ZMBME2JQQQ	1598	-			2012	SE	1
CC 5	ST 228	t001	TO2MBMDMGMK	385	I			2012	SW	2
	^-^	t688	TJMBMK	176	-			2008	SU	1
	^-^	t688	TJMBMK	176	-			2012	SW	1
	ST 5	t688	TJMBMK	176	VI			2012	SW	1
CC 6	ST 6	t14480	YC2FMBQBBQBLO	1592	IVa			2012	SW	1
	ST 6	t304	YC2FMBQBLO	91	-			2008	SN, U	2
CC 8	ST 8	t008	YHGFMBQBLO	1	IVa			2008	B, P, S, SV	6
	^-^	t008	YHGFMBQBLO	1	-			2012	SE, SW, U	3
	ST 8	t008	YHGFMBQBLO	1	VI			2012	SW	1
	ST 8	t2229	YHGFO	943	VI			2012	SW	2
	ST 8	t4229	YHGFMBH5BLO	1222	IVa	+		2012	SW	1
CC 15	^-^	t084	UJGBBGGJAGJ	21	-			2008	P, SW	3
	ST 2434	t084	UJGBBGGJAGJ	21	-			2012	B, P, SW, U	4
	^-^	t346	UJGBGGJAGJ	155	-			2012	SW	2
CC 22	ST 737	t005	TJEJNCMOMOKR	113	-		+	2008	SN	1
	ST 22	t005	TJEJNCMOMOKR	113	-	+		2012	B, SE, SW	5
	^-^	t005	TJEJNCMOMOKR	113	-			2008	B, P, S, SA, SE, SU, SV, U	13
	ST 22	t223	TJEJCMOMOKR	998	IVa	+	+	2012	SW	1
	-	t223	TJEJCMOMOKR	998	IVa		+	2008	B, P, Sp, SN, U	13
	-	t223	TJEJCMOMOKR	998	IVa		+	2012	B, SE, SW, U	15
	ST 22	t223	TJEJCMOMOKR	998	IVa			2008	DV, P	2
	-	t223	TJEJCMOMOKR	998	IVa			2012	SE	1
	ST 22	t223	TJEJCMOMOKR	998	V		+	2008	SU	1
	ST 22	t223	TJEJCMOMOKR	998	-		+	2008	P, SS, U	3
	ST 22	t309	TJCMOMOKR	66	IVa		+	2012	SE, SW	2
	ST 22	t541	TMOMOKR	846	IVa		+	2012	SW	2
	ST 22	t5485	TJEJCMOKKR	1601	IVa		+	2008	SW, U	2
	ST 22	t7063	TJEJCLMMOKR	1599	IVa		+	2008	SW	2
	ST 22	t7063	TJEJCLMMOKR	1599	V		+	2008	P, SS, SW, U	8
CC 30	ST 30	t012	WGKAKAOMQQ	33	-		+	2012	SE	1
	^-^	t019	XKAKAOMQ	19	-	+		2008	P, SW	2
	ST 30	t019	XKAKAOMQ	19	-	+		2012	SW	1
	ST 30	t021	WGKAKAOMQ	43	-	+		2008	P, DN, DU, SN, SS, U	16
	^-^	t021	WGKAKAOMQ	43	-	+		2012	SW	1
	^-^	t021	WGKAKAOMQ	43	-			2012	SW	2
	^-^	t096	WGKKAMQ	1591	-	+		2008	U	1
	ST 30	t096	WGKKAMQ	1591	-	+		2012	SW	1
	ST 30	t253	WGKAKAOMQQQQ	204	-		+	2008	B, P, U	5
	ST 30	t318	WGKKAKAOMQ	251	IVa	+		2008	B, DU, P, SA	4
	-	t318	WGKKAKAOMQ	251	IVa	+		2012	P, SW	6
	^-^	t318	WGKKAKAOMQ	251	-	+		2012	SW	3
CC 34	ST 34	t166	ZZ2PNGKBKGOLB	295	-		+	2012	SW	1
CC 80	ST 80	t044	UJGBBPB	70	IVc	+	+	2012	SW	1
	ST 80	t044	UJGBBPB	70	IVc			2008	B, P, Sp, SE, SN, SS, SV, U	23
	-	t044	UJGBBPB	70	IVc			2012	SE, SW, U	12
	ST 80	t458	T	1269	IV	+		2012	SW	1
CC 88	ST 88	t4103	TGFMBBBBPB	1270	-			2012	SW	3
CC 97	ST 97	t2112	TJGFMBBBBPB	1135	-			2008	DU	1
CC 121	ST 121	t314	XMJH2M	454	-	+		2008	P, SA, Sp	3
	^-^	t7793	XMJQM	1594	IV	+		2012	SW	1
	ST 121	t7793	XMJQM	1594	-	+		2012	SW	1
CC 133	ST 133	t1166	D2KFMJEMMMJQ	1593	-			2012	SW	2
CC 239	ST 239	t037	WGKAOMQ	3	III		+	2008	P	1
	ST 239	t037	WGKAOMQ	3	III			2008	B, CSF, P, SA, SS, U	7
	ST 239	t037	WGKAOMQ	3	III-Hg ^*c*^			2012	SW	1
CC 291	ST 291	t1149	XKBQBMM	718	-			2012	SW	3
	^-^	t1614	XKBQBBMMM	117	-			2012	SW	1
CC 398	ST 580	t6527	UAOAOQOMO	1600	-			2008	SW	1
CC 1153	ST 1153	t903	TLHMMDMG	824	I	+		2012	SW	1
	^-^	t903	TLHMMDMG	824	-	+		2012	SW	1

*S*. *aureus* isolates are classified hierarchically in the following order, from left-to-right: MLST clonal complex, MLST sequence type, *spa* type (both Ridom and eGenomics), SCC*mec* type, presence of genes encoding PVL (*lukF/S-PV*) and/or TSST-1 (*tst*), and year of isolation. Clonal complexes were inferred from *spa* repeat analysis and MLST typing of representative isolates of each genotypic pattern (except as noted). “No. of isolates” refers to the number of strains with the characteristics described in the preceding columns. CC, clonal complex; MLST, multilocus sequence typing; PVL, Panton-Valentine leukocidin; ST, sequence type; *spa*, staphylococcal protein A; SCC*mec*, staphylococcal cassette chromosome *mec*; TSST-1, toxic shock syndrome toxin.

^a^Multilocus sequence typing (MLST) was performed for one representative isolate from each clonal complex. For each row where a sequence type (ST) value appears, only one isolate was chosen, regardless of the number of isolates listed in the final column. A dash (-) indicates that MLST was not performed.

^*b*^A dash (-) indicates that no SCC*mec* targets were detected, and the strain is methicillin-susceptible (MSSA).

^c^“III-Hg” refers to SCC*mec* type III with SCC*mercury* (*ccrC*).

^d^Clinical isolate sources are abbreviated as follows: B, blood; CSF, cerebrospinal fluid; DN, nipple discharge; DU, urethral discharge; DV, vaginal discharge; P, pus; S, semen; Sp, sputum; SA, abscess swab; SE, ear swab; SN, nasal swab; SS, skin swab; SU, umbilical swab; SV, vaginal swab; SW, wound swab.

### *spa* typing and MLST

**Fig 2:**
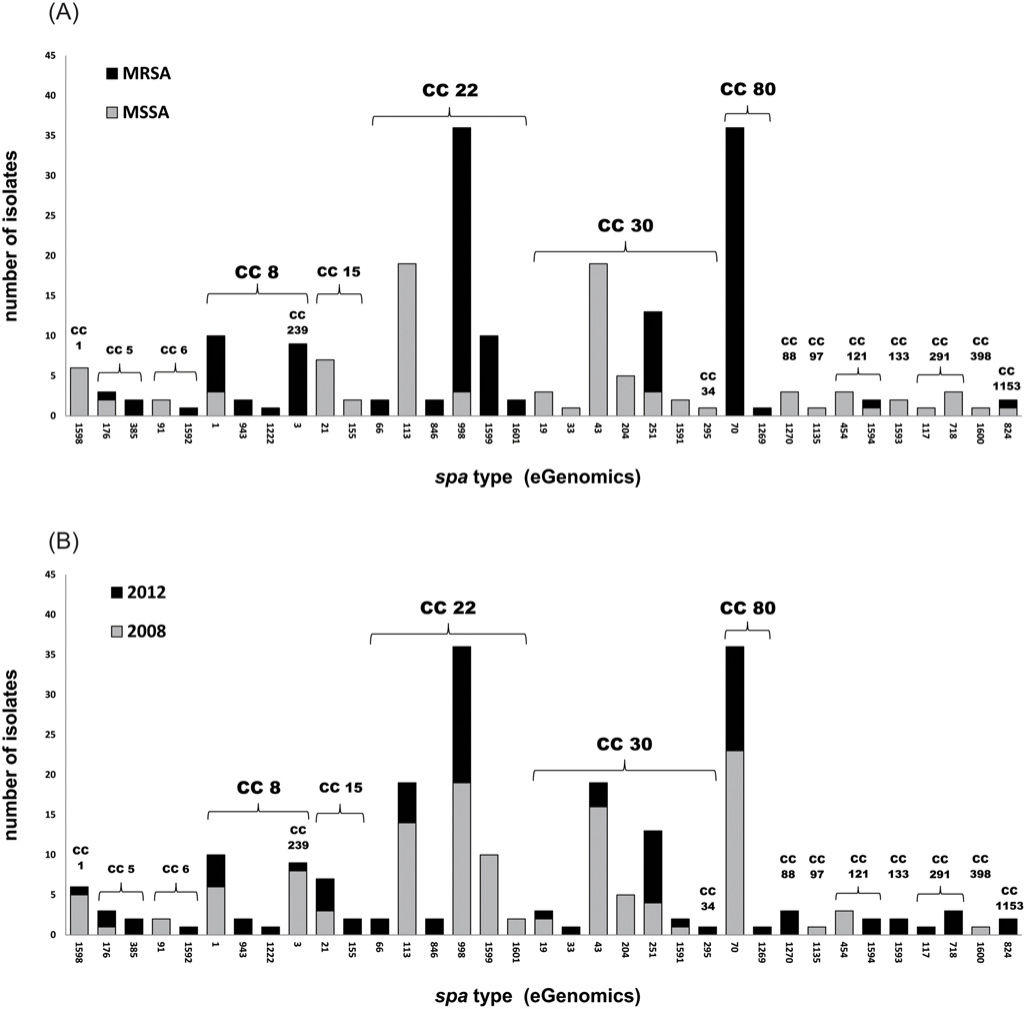
Relative frequencies of *spa* types among *S*. *aureus* isolates in this study. Vertical bars denote the relative frequencies of 35 *spa* types observed among *S*. *aureus* isolates in this study. a. Black bars denote *spa* types observed among MRSA isolates (*n* = 17); gray bars denote *spa* types observed among MSSA isolates (*n* = 24). b. Gray bars denote *spa* types observed among isolates from 2008 (*n* = 19); black bars denote *spa* types observed among isolates from 2012 (*n* = 28). eGenomics *spa* type numbers are depicted (corresponding Ridom *spa* types are listed in Table 1). *Spa* types are grouped together by MLST clonal complex; subgroups CC239 and CC34 are grouped with their parent complexes (CC8 and CC30, respectively). CC, clonal complex; MLST, multilocus sequence typing; *spa*, staphylococcal protein A.

A total of 35 unique *spa* types were detected among the 215 isolates (Table 1). As shown in Fig. 2, 11 *spa* types were associated exclusively with MRSA, 18 exclusively with MSSA, and 6 with both MRSA and MSSA. The *spa* type diversity was greater in 2012 than 2008, with 28 total *spa* types observed in 2012, *vs*. only 19 in 2008. The most prevalent *spa* types overall were *spa* 998 (Ridom t223) and *spa* 70 (Ridom t044), each of which accounted for 16.7% (36/215) of the total number of isolates. The prevalence of *spa* 998 (t223) increased from 15.1% (19/126) in 2008 to 19.1% (17/89) in 2012, while that of *spa* 70 (t044) decreased from 18.3% (23/126) in 2008 to 14.6% (13/89) in 2012.

*Spa* typing data were used to infer MLST clonal complexes (CC) for the majority of isolates, with MLST performed on 42 representative isolates for confirmation (and identification of unknown *spa* repeat patterns). A total of 17 different clonal backgrounds were inferred, 2 of which were associated exclusively with MRSA (CC80 and 239), 8 of which were associated exclusively with MSSA (CC1, 15, 34, 88, 97, 133, 291, and 398), and 7 of which were associated with both (CC5, 6, 8, 22, 30, 121, and 1153). Overall, CC22, CC30 and CC80 accounted for 33.0%, 20.0% and 17.2% of all *S*. *aureus* isolates, respectively, with slightly lower frequencies for each in 2012 than 2008. By contrast, the proportion of MRSA within CC22 and CC30 increased in 2012 (80.8% and 40.0%, respectively) compared to 2008 (62.2% and 14.3%, respectively). This was primarily associated with the decrease of MSSA-associated *spa* type 113 (t005) within CC22, and the increase in MRSA-associated *spa* type 251 (t318) within CC30. All CC80 isolates were MRSA, and were associated with *spa* type 70 (t044) and SCC*mec* type (IVc), except for one isolate with atypical *spa* type 1269 (t458).

As mentioned, the overall frequency of CC22 decreased slightly from 35.7% (45/126) in 2008 to 29.2% (26/89) in 2012, but there were notable changes in the strains mapped to this clonal complex over the four-year period. Among 79 strains classified as CC22, there were a total of eight different *spa* types, all identified as ST22 by MLST (with the exception of a single strain that typed as ST737, a single locus variant). Within the 2008 CC22 isolates, four different *spa* types were observed, with *spa* 113 (t005) and *spa* 998 (t223) accounting for 31.1% and 42.2% of these, respectively. Within the 2012 CC22 isolates, four different *spa* types were also observed, with *spa* 998 (t223) accounting for 64.7% of these; by contrast, the frequency of *spa* 113 (t005) was much lower in 2012 (14.7%), while *spa* 1599 (t7063) was not detected. All of the *spa* 113 (t005) isolates, and three of the *spa* 998 (t223) isolates, were MSSA; the remainder of the CC22 isolates were MRSA.

### PVL and TSST-1 detection

The genes coding for PVL (*lukF/S-PV*) were detected in 40.0% of all isolates, with overall prevalence increasing from 38.9% (49/126) in 2008 to 41.6% (37/89) in 2012. PVL-positive isolates were identified in six different clonal complexes (Table 1), including CC8, 22, 30, 80, 121, and 1153, with *spa* 251 (t318) CC30 isolates and *spa* 70 (t044) CC80 isolates being exclusively PVL-positive. Notably, PVL genes were not detected among any of the *spa* 1 (t008) CC8 isolates. The TSST-1 gene (*tst*) was detected in 27.4% of all *S*. *aureus* isolates, with overall prevalence decreasing from 28.6% (36/126) in 2008 to 25.8% (23/89) in 2012, and significantly higher frequency (*p* < 0.0001) among MRSA (39.7%) than MSSA (11.7%). Although identified in a number of isolates typed to CC30 (*n* = 6), CC34 (*n* = 1), CC80 (*n* = 1), and CC239 (*n* = 1), TSST-1 genes were associated with 70.4% (50/71) of all CC22 strains, and 91.7% (33/36) of all *spa* 998 (t223) strains. Notably, genes for both PVL and TSST-1 were observed in one *spa* 998 (t223) CC22 isolate and one *spa* 70 (t044) CC80 isolate, both from the 2012 collection.

A total of 17 *tst*-positive strains were selected in order to assay for TSST-1 toxin production, including 13 CC22 strains encompassing all observed *spa*, SCC*mec*, and PVL genotypic combinations; two representatives of each *tst*-positive CC30 *spa* type; and each of the singleton CC80 and CC239 *tst*-positive strains. Production of TSST-1 was determined *via* reverse passive latex agglutination (RPLA) as per the manufacturer’s instructions. All of the strains tested were positive for TSST-1 production, including those which also harbored genes for PVL.

### SaPI characterization

In order to better characterize the *tst*-positive’Gaza strain’, the SaPI elements from three *tst*-positive *spa* 998 (t223) strains were sequenced, including two MRSA (from 2012) and one MSSA (from 2008). Long PCR yielded a 10.5 kb amplicon using primer set intF1 and tst-Rv, and the resulting *tst*-bearing SaPI elements were completely sequenced and compared against the NCBI genome database. All three SaPI elements were identical, while BLAST results indicated >99.9% similarity to the sequence of SaPI2 from *S*. *aureus* “Harrisburg” strain RN3984 [49], with only 3 nucleotide differences (SNPs) within 10,578 bp. As described in the Methods, one of the three SaPI element sequences (from strain BK 38088) was submitted to GenBank (accession number KP334121).

## Discussion

The Palestinian Territories consist of two discontinuous areas: a) the West Bank, which shares a large border with Israel and Jordan; and b) the Gaza Strip, a small territory on the Mediterranean coast which shares most of its border with Israel, except for an 11-km southern border with Egypt. Since 2006, Gaza has been a semi-autonomous region, subject to border and travel restrictions enforced by Israel and Egypt. Although residents of Gaza occasionally travel to Israel for advanced medical care, Egypt is the primary portal for travel to the outside world, consisting mainly of students and foreign workers. The Egyptian border crossing in Rafah often serves as a portal for movement of people and goods, but has been increasingly subject to closure recently. As a result, the population of Gaza is somewhat more isolated than that of the West Bank, and may therefore be considered a “semi-closed” population [50].

Data regarding *S*. *aureus* and MRSA within the Palestinian Territories are generally scant [4,35]. Previous reports have mostly focused on Northern Palestine [34,51] and the West Bank [33], with relatively little detail regarding molecular epidemiology. Recently, a joint Israeli-Palestinian community-based nasal carriage study addressed the epidemiology of *S*. *aureus* in Gaza for the first time, highlighting the widespread presence of a CC22 clone dubbed the ‘Gaza strain’[36], which was subsequently described in two reports from Sicily, Italy [28,29]. In this study, we describe the molecular characteristics of clinical isolates collected from the primary tertiary-care hospital in Gaza in 2008 and 2012. We observed a similarly high prevalence of CC22, including a clone with the same *spa* type (998/t223) as the aforementioned Gaza strain. Moreover, we detected unusually high frequencies of CC22 isolates that harbor the gene (*tst*) coding for toxic shock syndrome toxin (TSST-1). To the best of our knowledge, this is the first report describing the molecular epidemiology of *S*. *aureus* clinical infection in the Gaza Strip.

The overall MRSA prevalence in this study was 56.3%, representing an increase of 3.6% from 2008 (54.8%) to 2012 (58.4%). Nosocomial MRSA prevalence in the Eastern Mediterranean was previously described as the highest in the Mediterranean region, with overall frequencies ranging as high as 52% (Egypt), 55% (Cyprus), and 56% (Jordan) [52]. Recent studies in Lebanon [53], Israel [54], Saudi Arabia [27] and Jordan [55] have noted nosocomial MRSA rates of 30%, 35%, 50%, and 62% respectively, while the only nosocomial report to date from Palestine (West Bank) described a MRSA prevalence of only 8.7% [51]. All other reports of MRSA prevalence in the Palestinian Territories have involved nasal carriage rather than infection [33,34,36], although notably, 45% of the *S*. *aureus* isolates from the aforementioned Gaza carriage study were methicillin-resistant [36]. Therefore, the MRSA frequencies described in this report may be considered among the highest described in the region thus far.

Molecular analysis revealed a surprising degree of genotypic diversity. A total of 35 different *spa* types corresponding to 17 MLST clonal complexes were identified, with *spa* type diversity increasing from 2008 to 2012. Consistent with the *S*. *aureus* literature, MRSA strains showed less genetic diversity than MSSA, and as observed in the carriage study from Gaza [36], CC22 was the predominant lineage, with *spa* 998 (t223) accounting for half (50.7%) of the CC22 isolates. Interestingly, much more diversity was found in the current hospital-based study than in the carriage study, possibly due to the longer sampling period in our study. Whereas *spa* 998 (t223) was the only CC22 *spa* type found in the carriage study, accounting for 64% of all MRSA isolates, in this study a total of six different CC22 *spa* types were identified, with *spa* 998 (t223) accounting for only 27.3% of all MRSA isolates. Moreover, whereas the carriage study only identified three additional MRSA clonal backgrounds (CC5, 78, and 80) and four MSSA clonal backgrounds (CC15, 22, 45, and 291), the present study identified nine MRSA-associated clonal backgrounds (CC5, 6, 8, 22, 30, 80, 121, 239, and 1153) and fifteen MSSA-associated clonal backgrounds (CC1, 5, 6, 8, 15, 22, 30, 34, 88, 97, 121, 133, 291, 398, and 1153). Nevertheless, the high prevalence of the Gaza strain in the carriage study argues in favor of a community-based origin [36], and suggests that the high levels of MRSA carriage (12–13%) observed in the community may serve as a reservoir for the nosocomial CC22 lineages described in our study [25].

Notably, the predominant clonal backgrounds and *spa* types observed in both Gaza studies differ markedly from those described in the West Bank and Israel. Previous reports from the West Bank have identified CC1 and CC5 [33], as well as CC239 [56], while reports from Israel have described CC5 [54,57], CC8 [54,57], and CC45 [57,58] as the dominant clonal backgrounds. Unfortunately, molecular epidemiologic data from Egypt are exceedingly rare, consisting of two studies featuring small numbers of PVL-positive CA-MRSA isolates from lineages also found in this study [59,60], and, more recently, a nasal carriage study involving outpatients in Egypt and Saudi Arabia [61]. By contrast, more detailed studies from Lebanon [2,32,53] and Jordan [24,25,32,55,62] reveal a number of *spa* types and clonal backgrounds also detected in this study. The contrast between the molecular epidemiology of *S*. *aureus* in Gaza and Israel is noteworthy, given that residents of Gaza frequently seek advanced medical care within Israel. Interestingly, whereas SCC*mec* types I, II, and V are common in Israeli hospitals [7,54], types I and V are relatively uncommon in Gaza, while type II was not detected in this study.

Biber *et al* [36] defined the Gaza strain (which accounted for 64% of all MRSA isolates in their study) as ST22, *spa* 998 (t223), SCC*mec* IVa, PVL-negative, and genetically distinct from the pandemic EMRSA-15 clone (as demonstrated by PFGE); however, the authors did not address whether it harbored the TSST-1 gene. In this study, although a total of 49/121 (40.5%) MRSA isolates belonged to CC22, only 31/121 (25.6%) possessed all of the aforementioned characteristics. In addition to these, one SCC*mec* IVa-associated *spa* 998 (t223) isolate was PVL-positive; one *spa* 998 (t223) isolate harbored SCC*mec* V; and three *spa* 998 (t223) isolates were MSSA. Notably, however, all but three of the *spa* 998 (t223) isolates in this study harbored TSST-1 genes, including the PVL-positive isolate. Although most CC22 strains (including EMRSA-15) do not harbor TSST-1 [63], *tst*-positive CC22 strains have nevertheless been described in the literature. Most recently, 8/10 (80.0%) nasal MRSA isolates obtained in 2013 from healthy preschool children in Palermo, Italy were characterized as *tst*-positive, *spa* 998 (t223), and SCC*mec* IVa, while 5/74 (6.8%) MSSA isolates were also *spa* 998 (t223) [28]. In an earlier study, the authors detected the same strain in 166/187 (88.8%) MRSA-colonized infants in a neonatal intensive care unit (NICU), describing it as “endemic” [28]. In both studies, the authors refer to the *tst*-positive strain as UK-EMRSA-15/“Middle Eastern Variant”, and suggest it is identical to the ‘Gaza strain’ described by Biber *et al* [36].

Within the Middle Eastern region, 2/16 (12.5%) EMRSA-15 isolates from Kuwaiti hospitals [26]; 4/21 (19.0%) CC22 isolates from a hospital in Abu Dhabi [23]; 6/30 (20.0%) CC22 isolates from a hospital in Riyadh (Saudi Arabia) [27]; and 5/5 (100.0%) *spa* 998 (t223) isolates from a community-based carriage study in Jordan [24] were all identified as *tst*-positive, while a more recent study in Jordan identified *tst*-positive *spa* 998 (t223) as the predominant MRSA strain among nasal isolates from healthcare workers and community-based individuals [25]. As of this writing, a carriage study in Egypt and Saudi Arabia also identified *spa* 998 (t223) in nasal isolates from outpatients, but presence of *tst* was not addressed [61]. Meanwhile, a large study in southern India uncovered 6 clinical and 7 nasal EMRSA-15 isolates which were all positive for both PVL and TSST-1 [3]. Similarly, isolates from 2005–2007 referred to the national Staphylococcal Reference Laboratory in the United Kingdom included five CC22 strains harboring both TSST-1 and PVL [30]. Lastly, the first report of an EMRSA-15 variant in the United States involved a *tst*-positive isolate from a 10-month old infant which was likewise described as *spa* 998 (t223), SCC*mec* IV, and PVL-negative [31].

In this study, TSST-1 genes were also detected in single isolates of CC80 and CC239, suggesting that CC22 strains may be serving as a TSST-1 reservoir for other clonal backgrounds in Gaza. The CC80 isolate also harbored PVL, and the presence of both genes in an isolate corresponding to the Gaza strain suggests the potential for greater numbers of isolates harboring genes for both toxins. These observations are supported by the detection of both PVL and TSST-1 genes in 3/5 *spa* 998 (t223) isolates obtained from Shohada Al-Aqsa Hospital in Gaza during the same collection period in 2012 (data not shown). Moreover, in a recent regional survey which identified TSST-1 genes in 18/31 (58.1%) CC80 strains from Jordan, 7 also harbored PVL genes [32]. As the authors of that study note, while carriage of both PVL and TSST-1 is rare in *S*. *aureus*, a previous study from Jordan also identified two CC80 isolates harboring the genes for both toxins [24]. Likewise, the aforementioned studies from India [3] and the U.K. [30] also observed simultaneous carriage within several clonal backgrounds, including CC22. Although the clinical implications of harboring both toxins are unclear at this time, a recent study suggests they may not be expressed at similar levels during infection [64].

Our study has several limitations. As mentioned earlier, very little clinical and demographic data were available, thereby limiting the possibility of epidemiological inference, including a clear distinction between healthcare-associated (HA) vs. community-associated (CA) infections. Likewise, morbidity and mortality data was not available, leaving the clinical implications of PVL- and TSST-1 bearing strains unresolved. Future studies should aim to further characterize TSST-1 harboring CC22 clones, in Gaza as well as other countries where they have been reported, in order to discern whether such strains are spreading internationally, or whether CC22 strains are acquiring TSST-1 independently. A recent phylogenomic analysis of 193 spatio-temporally diverse strains detailed the population structure of CC22, clearly distinguishing EMRSA-15 from various other lineages. However, strains from the Middle East were not included in the study, while only two MSSA strains were observed to harbor TSST-1, highlighting the unique nature of the Gaza strain among known CC22 lineages [63].

Given the pandemic spread of EMRSA-15, the potential for acquisition of TSST-1 from CC22 strains such as the ones described in this study is of significant clinical concern. Moreover, in light of recent reports from Sicily [28,29], surveillance programs should endeavor to monitor spread of the Gaza strain to other regions [25], as well as the spread of TSST-1 to other clonal backgrounds, and further occurrence of both PVL and TSST-1 within the same strain. Lastly, clinical epidemiology studies should be undertaken to determine risk factors, transmission patterns, and clinical outcomes for CC22 strains harboring TSST-1 [25], given the high rate of endemicity observed in multiple studies including this report [25,28,29]
